# Integration of RNA Editing with Multiomics Data Improves Machine Learning Models for Predicting Drug Responses in Breast Cancer Patients

**DOI:** 10.21203/rs.3.rs-5604105/v1

**Published:** 2024-12-17

**Authors:** Yanara A. Bernal, Alejandro Blanco, Karen Oróstica, Iris Delgado, Ricardo Armisén

**Affiliations:** Universidad del Desarrollo; Universidad del Desarrollo; Universidad de Talca; Universidad del Desarrollo; Universidad del Desarrollo

**Keywords:** machine learning, multiomic integration, RNA editing, ADAR, breast cancer, drug response, prediction, random forest

## Abstract

**Background:**

The integration of conventional omics data such as genomics and transcriptomics data into artificial intelligence models has advanced significantly in recent years; however, their low applicability in clinical contexts, due to the high complexity of models, has been limited in their direct use inpatients. We integrated classic omics, including DNA mutation and RNA gene expression, added a novel focus on promising omics methods based on A>I(G) RNA editing, and developed a drug response prediction model.

**Methods:**

We analyzed 104 patients from the Breast Cancer Genome-Guided Therapy Study (NCT02022202). This study was used to train (70%) with 10-fold cross-validation and test (30%) the drug response classification models. We assess the performance of the random forest (RF), generalized linear model (GLM), and support vector machine (SVM) with the Caret package in classifying therapy response via various combinations of clinical data, tumoral and germline mutation data, gene expression data, and RNA editing data via the LASSO and PCA strategies.

**Results:**

First, we characterized the cohort on the basis of clinical data, mutation landscapes, differential gene expression, and RNAediting sites in 69 nonresponders and 35 responders to therapy. Second, regarding the prediction models, we demonstrated that RNA editing data improved or maintained the performance of the RF model for predicting drug response across all combinations. To select the final model, we compared the F1 score between models with different data combinations, highlighting an F1 score of 0.96 (95% CI: 0.957--0.961) and an AUC of 0.922, using LASSO for feature selection. Finally, we developed a nonresponse risk score on the basis of features that contributed to the selected model, focusing on three RNA-edited sites in the genes KDM4B, miRNA200/TTLL10-AS1, and BEST1. The score was created to facilitate the clinical translation of our findings, presenting a probability of therapy response according to RNA editing site patterns.

**Conclusion:**

Our study highlights the potential of RNA editing as a valuable addition to predictive modeling for drug response in patients with breast cancer. The nonresponse risk score could represent a tool for clinical translation, offering a probability-based assessment of therapy response. These findings suggest that incorporating RNA editing into predictive models could enhance personalized treatment strategies and improve decision-making in oncology.

## Introduction

The integration of artificial intelligence (AI) into clinical decision-making holds immense promise for enhancing health outcomes across diverse diseases, including cancer [1]. AI tools have demonstrated potential in early diagnosis, comprehending intricate biological mechanisms, and facilitating the development of novel therapeutic strategies [2]. However, the successful translation of AI models into clinical practice for cancer treatment faces substantial challenges.

The heterogeneity of the response to anticancer drugs and the development of therapeutic resistance represent significant clinical challenges, leading to increased mortality rates worldwide [3, 4]. Resistance to therapy in BC is multifactorial, with contributing mechanisms including increased drug efflux, alterations in the tumor microenvironment, epithelial–mesenchymal transition, tumor heterogeneity, therapeutic target alterations, adaptive responses, and DNA damage repair [4–7]. Between 30% and 50% of BC patients may develop therapy resistance, resulting in a drastically reduced survival time of 2 to 3 years compared with 5 years in responders [8]. Therefore, early prediction of the therapeutic response is crucial for timely and effective clinical decision-making.

Recent advancements have integrated conventional omics data, such as germline and tumoral DNA mutation data and RNA expression data, into AI drug response models. Nevertheless, these approaches often overlook crucial factors influencing tumor complexity. This limitation, compounded by methodological issues such as poor data quality, missing data, and small sample sizes, contributes to the difficulty in replicating cancer study findings across independent cohorts [9]. These factors introduce biases into AI predictive models, complicate the interpretation of machine learning’s “black box” concept, and hinder the translation of AI models into clinical practice [10, 11].

In this context, RNA editing, a posttranscriptional modification mediated by ADAR enzymes, presents a promising avenue to address some of these challenges. This process, involving the conversion of adenosine to inosine (A > I(G)) in RNA, can significantly impact gene product structure and function, influencing tumor biology and drug response [12]. Despite its potential relevance, research on RNA editing in cancer, particularly its integration into AI models, remains limited. While pancancer studies have described certain RNA edited sites [13] and preliminary work has explored the role of RNA editing in the drug response of patients with breast cancer (BC) via cell lines [14, 15], its clinical implications remain largely unexplored. Notably, RNA editing has not been systematically incorporated into AI models for predicting clinical outcomes in cancer [16].

Recent studies have highlighted the potential of RNA editing-based predictive models in various cancers, including gastric cancer [17], lung cancer [18], acute myeloid leukemia (AML) [19], and lower-grade gliomas [20]. However, the utilization of RNA editing for predicting drug response in BC remains underexplored.

This study addresses these critical gaps by integrating multiomics data, with a novel focus on A > I(G) RNA editing, to enhance the prediction of drug response in BC. We leverage clinical trial data to develop a machine learning-based risk score for nonresponse and a probability-based score to assess the likelihood of therapy response, with the ultimate goal of more precise and actionable clinical decision-making in BC, improving patient outcomes.

## Methods

### Dataset and breast cancer patients

One hundred and four patients were analyzed from the Breast Cancer Genome-Guided Therapy Study (ClinicalTrials.gov: NCT02022202) out of one hundred and eighteen BC patients according to data availability. The clinical characterization of these patients was based on therapy response, which was defined as a response to therapy when reported as a pathological complete response in the breast and nodes (path-CR) after 24 weeks of chemotherapy (adriamycin and cyclophosphamide or epirubicin and cyclophosphamide or 5-flurouracil, epirubicin and cyclophosphamide), whereas nontherapy response referred to when there was no pathological complete response [21, 22]. Additionally, the molecular subtype was defined based on baseline Ki67 results, estrogen receptor levels, and HER2 status (by immunohistochemistry (IHC) or fluorescence in hybridization (FISH)) from the original study.

### Whole Exome Sequencing (WES) Analysis and Variant Calling

WES data from paired tumor and normal samples were analyzed via an automated pipeline deployed on the SevenBridges cloud platform (https://www.sevenbridges.com/). The raw sequencing reads in FASTQ format underwent initial processing with Trim Galore [23] to remove low-quality bases and adapter sequences, ensuring high-quality input for downstream analysis. The trimmed FASTQ files were then converted into unmapped BAM (uBAM) format via Picard’s FatsqToSam tool [24], which added the read group information necessary for alignment. The uBAM files were subsequently aligned to the GRCh38 reference genome via BWA-MEM [25]. Following alignment, the BAM files were processed following GATK Best Practices [26–28] to produce high-quality analysis-ready BAM files. This included marking duplicate reads with Picard’s MarkDuplicates to mitigate biases from PCR amplification and performing base quality score recalibration (BQSR) via GATK’s BaseRecalibrator and ApplyBQSR, incorporating known variant sites to ensure accuracy.

Somatic variants were identified via GATK Mutects2 in tumor-normal mode. The matched normal samples were utilized to distinguish somatic mutations from germline variants and sequencing artifacts. GATK’s FilterMuectCalls was applied to refine the somatic variant calls further. Germline variants were called via GATK’s HaplotypeCaller in GCVF mode on the normal samples. The resulting gVCFs were combined via CombineGVCFs, and the joint genotyping step was performed with GenotypeGVCFs to produce a multisample VCF. The genotyped VCF was filtered using GATK’s VariantRecalibrator and ApplyVQSR separately for both SNPs and InDels. The VCFs were subsequently split into individual VCFs to facilitate downstream analyses.

Annotation of both somatic and germline variants was conducted via the Ensembl Variant Effect Predictor (VEP) [29], which adds functional and clinical information, including gene impact, variant consequences, and pathogenicity predictions. Finally, the annotated VCFs were converted into mutation annotation format (MAF) files via the vcf2maf tool [30] to enable compatibility with downstream analysis.

### RNA-seq analysis

The RNA sequencing data were preprocessed and analyzed via the nf-core/rnaseq pipeline (v3.14.0) implemented in NextFlow (v23.04.2). The analysis was performed with GRCh38 as the reference genome and followed standard best practices for RNA-seq data analysis. Initially, raw FASTQ files were subjected to quality control and adapter trimming via Trim Galore v0.6.7, ensuring that low-quality bases and adapter sequences were removed. Trimmed FASTQ files were then aligned to the reference via STAR in two-pass mode, which improves splicing accuracy by using junction information obtained from the first pass during the second pass of alignment. Salmon quantification was performed alongside STAR alignment to estimate transcript abundance via quasimapping and expression quantification. Gene annotation for alignment and quantification was based on the GENCODE v43 annotation file, ensuring compatibility with the reference genome. Multiple quality control steps were performed on the BAM files via RSeQC, SAMtools, Dupradar and Qualimap to ensure the integrity of the data. MultiQC [31] was used to report the results.

### Tumoral and germline DNA variant characterization

We considered tumor variants reported in genes listed in the Cancer Gene Census (CGC) from COSMIC [32]. We evaluated differences per variant and gene mutation in the responder and nonresponder groups via Fisher’s exact test for germline mutations and focused on genes related to high-risk cancer predisposition: ATM, BAPI, BMPR1A, BRCA1, BRCA2, BRIP1, MSH2, MSH6, MUTYH, DICER1, PALB2, RUNX1, SDHAF2, SDHB, SDHC, and SDHD as Tier 1 of high risk; Tier 2: APC, CDH1, MLH1, MEN1, NF1, NF2, PMS2, POLE, PTEN, PTPN11, RB1, RET, SMAD4, SMARCA4, STK11, TGFBR2, TSC1, TSC2, VHL and WT1 as intermediate risk; and Tier 3 BARD1, CHECK2, HNF1A, FH, NBN, RAD50, RECQL4, and TP53 [33].

### Gene expression abundance estimation

Differential expression analysis (DEA) between the response and nonresponse groups was performed via the raw transcript-level quantification files generated by Salmon during the nf-core/rnaseq analysis [34]. To enable the use of DESeq2 [35] for differential expression analysis, the transcript quantification values were approximated to the nearest integer. Gene annotations, including HGNC gene symbols, were retrieved by querying Ensembl Transcript IDs via biomaRt [36]. Differentially expressed transcripts were visualized via a volcano plot generated with EnhancedVolcano, applying a p-adjusted cutoff of < 0.05 and a fold-change (FC) threshold of > 2.5. For the creation of predictive models, Salmon’s gene-level quantification files normalized to transcripts per million (TPM) were utilized.

### High-confidence RNA editing identification

REDITools was used to identify RNA edited sites on the basis of a previously published methodology, which briefly consisted of BAM files from STAR alignment in nf-core/rna-seq [37, 38]. After applying REDITools to all the BAM files, we excluded all sites found as mutations A/G or T/C from the DNA variants called in the tumor and/or germline. For RNA-edited site identification, we consider only sites that are the reference/alternate of A/G or T/C. For these sites, we calculated the RNA editing level at each site, which consists of the ratio between mismatch (A/G on the positive strand or T/C on the negative strand) reads and total readings at the site (both mismatch and match, represented by A/A on the positive strand or T/T on the negative strand). The RNA edited level per site was included in the models. Additionally, we used RNA-editing tests (REDITs) to identify RNA-edited sites between responders and nonresponders via the beta-binomial distribution for characterization and selection of RNA-edited sites (FC cutoff > 0.05 and p adjust < 0.01) [39].

### Predictive models

We preprocessed the data of the study cohort, which was composed of one hundred and four patients from the Breast Cancer Genome-Guided Therapy Study. We included in the model only the selected features from each omics dataset (tDNA/gDNA, DES, DGE) and the most relevant clinical features (molecular subtype, histological type, TNM stage and age group). These features are the inputs of the drug response classification models. Once these features were assigned to each patient, we divided the preprocessed dataset into a training subset (70%) and a test subset (30%). A 10-fold cross-validation was applied to ensure the robustness of the model. We performed random forest (RF), generalized linear model (GLM), and support machine vector (SMV) analyses for the classification of response or nonresponse to therapy.

This is due to the high number of predictors in the input dataset (Fig. 2). For selecting features, we implemented two strategies. For principal component analysis (PCA), we selected PCAs with a variance explained in an elbow plot or least absolute shrinkage and selection operator (LASSO). With respect to model training, all classification models were subjected to tenfold cross-validation. We trained the models when we determined the best value for the hyperparameter to improve the accuracy, and we retrained the model, which was selected as the final model. For these models, we evaluated different combinations of features:

Model 1: Therapy response ∼ Clinical + Gene Expression (DGE)

Model 2: Therapy response ∼ Clinical + Gene Expression (DGE) + **RNA editing (DES)**

Model 3: Therapy response ∼ Clinical + tDNA/gDNA

Model 4: Therapy response ∼ Clinical + tDNA/gDNA + **RNA editing (DES)**

Model 5: Therapy response ∼ Clinical + Gene Expression (DGE) + tDNA/gDNA

Model 6: Therapy response ∼ Clinical + Gene Expression (DGE) + tDNA/gDNA + **RNA editing (DES)**

For the meta-analysis, we calculated metrics (accuracy, recall, precision and F1 score) that represent the performance of the models via the prediction of each model on the test dataset without the therapy response information. The metrics were calculated from a confusion matrix defined as follows:

$$Precision:TP\text{/}\left(TP+FP\right)$$

$$Recall:TP\text{/}\left(TP+FN\right)$$

$$F1-\text{score}\;:\;2x\;\left(\left(Precision\;x\;Recall\right)\text{/}\left(Precision+Recall\right)\right)$$

where TP is the true positive rate, TN is the true negative rate, FP is the false positive rate, and FN is the false negative rate. To calculate the confidence interval for the F1 score, bootstrapping was performed 1000 times. We selected the best model from the F1 score information. Finally, for the selected best model, we identified and ranked the most informative features determined by varImp from the Caret package in the final models.

### Design of a nonresponse risk score based on features from machine learning models

A risk score for nonresponse to drugs was created on the basis of the methodology published by Sullivan [40] to facilitate the translation of the model to clinical settings with the fewest possible features. Briefly, this approach involves estimating parameters from a multivariable logistic regression model. In this model, the β coefficient is taken in its absolute value and approximated to the smallest absolute value, which serves as the score for each variable. To calculate the total score, all individual scores are summed. Additionally, the probability of nonresponse is determined as:

$$1\text{/}e^{-\left(intercept+\beta \;*\;point\;total\right)}$$

where β corresponds to the smallest beta in the model.

## Results

### Clinical and molecular characterization of the training and testing cohorts

**Figure 1:**
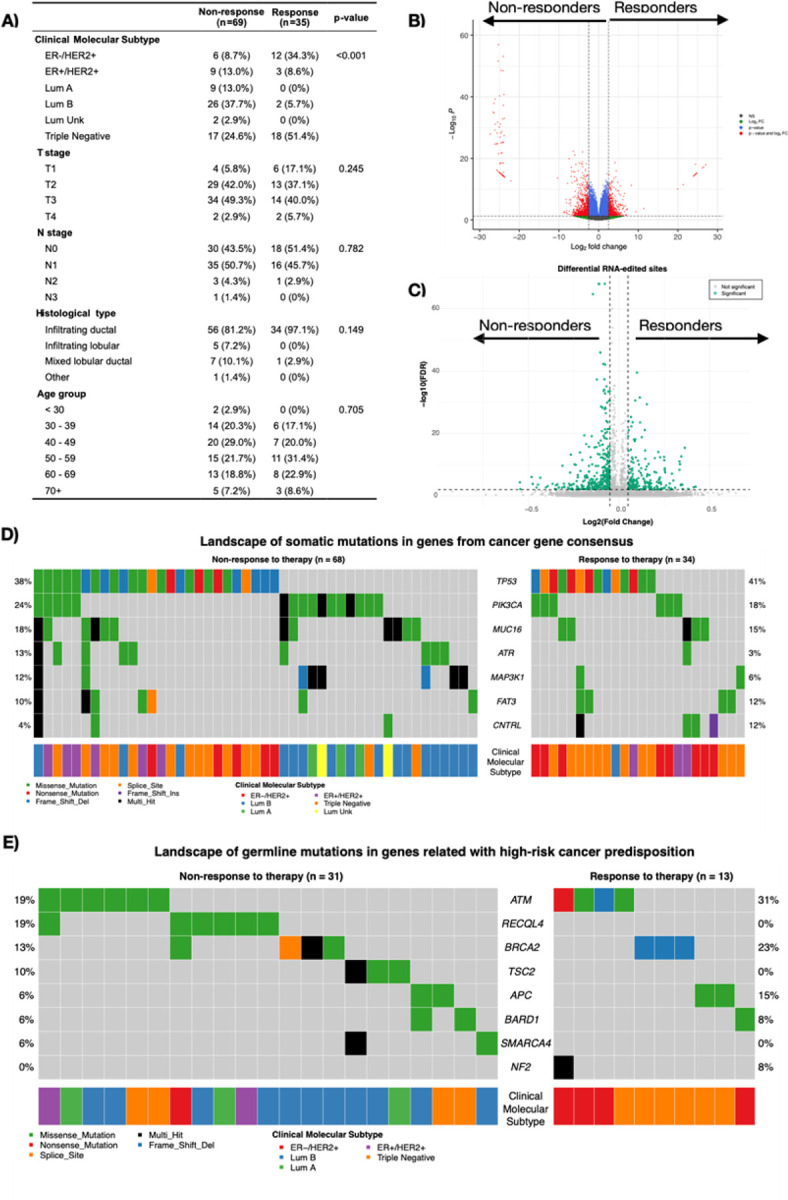
Clinical and molecular characterization of the training and testing cohorts. A) Table of clinical characterization by drug response; B) Differential expression transcript in Volcanoplot FC cutoff >2.5 and p-adjust cutoff <0.05; C) Differential RNA edited level in Volcanoplot FC cutoff >0.05 and p-adjust cutoff <0.01; D) landscape of somatic mutations in genes from the Cancer Gene Census (CGC) and E) landscape of germline mutations in genes related tohigh-risk cancer predisposition in Oncoplot by drug response, each row represents a gene, and each column represents a patient with at least one variant (n= 44 subjects). The colors indicate different types of mutations and the molecular subtypes of the patients.

The clinical characteristics analyzed included molecular subtype, tumor size, nodal status, histological type, and age group. Molecular subtype was significantly different between the groups (p < 0.001), whereas other variables, such as tumor size, nodal status, histological type, and age, were not significantly different (Fig. 1A). Through differential transcript expression analysis, we identified 996 differentially expressed genes (DEGs). We highlighted transcripts of SNX14, RHOT2, PIK3R1, SLC7A4, DTNA, and even RAD51 in the nonresponders, whereas genes such as IFITM3, CYP2T1P, TMUB2, and PAX6 were prominent in the responders (Fig. 1B). However, we did not find significant differences in ADAR1 (ENST00000492630.2) expression between the groups (Table S1). With respect to the differentially expressed RNA edited sites, 500 sites were identified as significantly different between responders and nonresponders to therapy (DES). Among these, we highlight specific sites within genes such as ALPL (COSV66379629), DHTKD1, ABCC4 (COSV65312135), GAA (COSV56406822), USP34, ZNF662, and NFKBIZ (COSV58198879). These sites result in missense mutations, have been previously reported in the COSMIC database, and are predicted to be potentially damaging by PolyPhen and deleterious by SIFT (Fig. 1C, Table S2).

Among the 290 somatic variants identified in the cancer gene consensus (CGC) cohort, the most altered genes in nonresponders were somatic variants in TP53, PIK3CA, and MUC16, whereas responders presented mutations in genes such as ATR, MAP2K1, and FAT3 (Fig. 1D). We did not find significant differences by gene or by variant between the responder and nonresponder groups. (Table S3–S4). In terms of germline mutations, we selected 47 variants from the list of high-risk cancer predisposition genes; only 44 patients (42.31%) had at least one germline mutation. Notably, responder patients presented alterations in genes such as ATM, RECQL4, and BRCA2, whereas APC and NF2 were prominent in nonresponders (Fig. 1E).

### Models of machine learning for drug response

**Figure 2:**
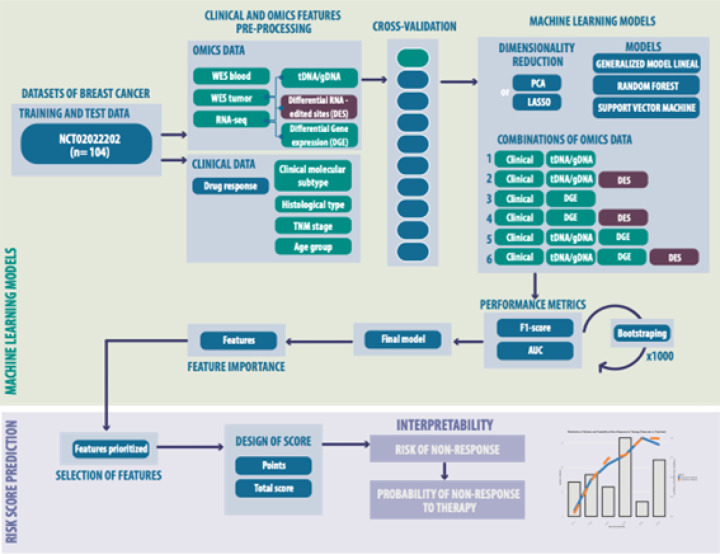
Design of a drug response risk score based on omics and clinical data via machine learning models. Scheme of workflow for predicting response to therapy via machine learning models.

We developed ML models to predict drug response in BC patients via clinical and omics data. Data from whole-exome sequencing (WES), RNA sequencing (RNA-seq), and clinical variables were preprocessed and reduced via PCA or LASSO. Models, including GLM, RF and SVM, were trained and tested on various omics data combinations, with performance evaluated via the F1 score and AUC through cross-validation and bootstrapping. Relevant features were identified to design a risk score for nonresponse to therapy, enabling interpretable predictions with a probability-based assessment of therapy outcomes (Fig. 2).

**Figure 3:**
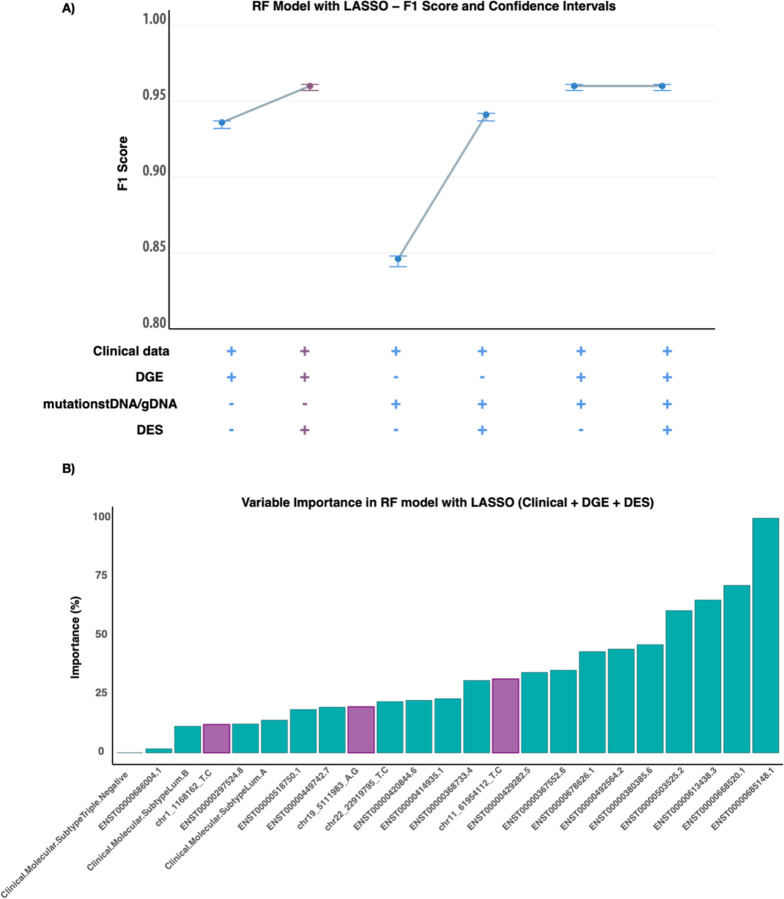
The use of RNA editing sites improved the performance of random forest with LASSO models in predicting therapeutic response. A) Summary of the F1 scores of all the models by RF with LASSO and B) important features of the selection of the final model.

The RF model incorporating clinical data, RNA editing, and gene expression data was selected via the LASSO variable selection technique, achieving an F1 score of 0.96 (95% CI: 0.957–0.961) (Fig. 3A), representing the best-performing model with fewer variables than the other models and data combinations (Figure S2). This pattern was also observed in the GLM and SVM models with LASSO. However, when PCA was used, the results were inconsistent across models, with some combinations showing improvement, others maintaining the same performance, and some experiencing a decrease in the F1 score (Figure S2). This selection also supports parsimony and facilitates the model’s translation to clinical settings. In all the RF models with LASSO, RNA editing was observed to enhance model performance (Fig. 3A). Similarly, we found that models including RNA editing sites presented improved F1 scores, suggesting that RNA edits provide key information for predicting therapy response.

We further analyzed the selected model, which includes 23 features, highlighting five RNA edited sites and sixteen transcripts (Fig. 3B). We detected the demethylation of histones via gene ontology analysis at the RNA editing level in five DESs (Figure S3), and sixteen DGE features strongly affected metal ion homeostasis and related cellular processes in the studied context (Figure S4).

From the selected model, we can prioritize the variables that contribute most significantly by analyzing their importance. The key contributors include ENST00000634769.1 (lnc-PCSK9-4:7), ENST00000613438.3 (CTCF-DT), ENST00000503525.2 (TCL1), ENST00000668520.1 (IL21-AS1-204), and ENST00000685148.1 (PLCB4-216), which are associated with molecular subtypes luminal A and B. Additionally, RNA-edited sites in KDM4B, miRNA200, and BEST1 further strengthen the model.

### Score of risk of nonresponse to therapy

We developed a risk score for nonresponse to therapy using the variables with the greatest contribution in the ML model. A logistic regression model was then employed to calculate beta coefficients and odds ratios. With this information, a weighted score was assigned to each variable, where the total sum generated a predictive risk index for nonresponse to therapy. However, most of the variable scores were near zero. Surprisingly, three edited sites presented high odds ratios and therefore had nonzero scores. The procedure was then repeated with these three variables to create the index, which had a maximum score of 50 points and a minimum of 0 points. The final point score was significantly lower in responders, with a mean of 16 (SD 15.4) points, than in nonresponders, who had a mean of 35.7 (SD 12.6) points (Figure S5).

To study the associations of features with nonresponse to therapy, we calculated the β coefficient, odds ratio (OR) and 95% confidence interval (95% OR), p value and points (Table 1). To interpret this index, the risk of nonresponse to therapy was calculated and aligned with the score obtained: a higher score indicates a greater risk of nonresponse to therapy, which is concordant with the therapy response observed and predicted by the score (Fig. 4A). The combined index demonstrated superior performance, achieving an area under the curve (AUC) of 0.823, indicating a high predictive ability to differentiate between responders and non-responders (Fig. 4B). Among individual features, the chr19_5111983_A/G variant achieved an AUC of 0.710, the chr11_61954112_T/C variant obtained an AUC of 0.705, and the chr1_1168162_T/C variant presented an AUC of 0.684, all showing acceptable predictive capacity (Fig. 4C).

## Discussion

Our comprehensive clinical and molecular characterization of the training and testing subsets of BC patients has provided insights into the factors associated with drug response. We identified significant differences in clinical and molecular features between responders and nonresponders. The integration of RNA editing data provided valuable improvement, enhancing the performance of machine learning models, with the RF model achieving the best F1 score when LASSO-selected features were used. Our findings suggest that RNA editing sites, particularly those in KDM4B, miRNA200/TLL10-AS1, and BEST1, provide critical predictive information for therapy response. The risk score model we developed allows for the assessment of nonresponse risk, with higher scores correlating with increased risk. The score represents a step toward practical clinical application, providing a probability-based assessment of nonresponse to therapy.

The use of RNA editing sites improved the performance of most GLM, RF and SVM models in LASSO. DES with DGE and DES, DGE and tDNA/gDNA were the models with the highest performance. According to recent studies, independently, gene expression and variants in DNA have similar effects on drug response in breast cancer patients [41]. Additionally, when we trained the models with only clinical and DNA data, the F1 score was lower; these data support the decision concerning the final model of the DES and DGE data.

Our risk score of nonresponse to therapy on the basis of three RNA-edited sites achieved an AUC of 0.823. Similarly, another model for predicting the prognosis of lower-grade gliomas, which is based on four RNA-edited sites (PRKCSH chr19:11561032, DSEL chr18:65174489, UGGT1 chr2:128952084, and SOD2 chr6:160101723), also reported an AUC of 0.823 [20]. In BC, four RNA editing sites (ARSD A2874 > I, ZNF791 A2280 > I, MED18 A1552 > I, and RAD1 A1415 > I) were included in the assessment of survival prognosis. Although the study reported the C-index, a metric for survival prediction, it can be considered comparable to the AUC, as both evaluate discriminatory ability. The C-index was 0.742 in the testing cohort (n = 311) and 0.869 in the external cohort (n = 197) [42]. Additionally, a signature based on 35 RNA-edited sites had an AUC of 0.907 for predicting chemotherapy response [43]. Overall, across all studies, RNA editing-based models exhibited high performance. However, these models initially include only RNA editing data, and the potential improvement in predictive outcomes by incorporating epitranscriptomic data has not yet been described.

KDM4B disrupts the DNA damage repair (DDR) machinery, leading to cellular transformation and immortalization, a key step in cancer development [44]. Similarly, miRNA-200 plays a pivotal role in the drug response by regulating c-MYB expression, which affects epithelial–mesenchymal transition (EMT) and tamoxifen resistance in estrogen receptor-positive BC cells [45]. Notably, RNA editing of miRNA-200 has been proposed as a novel oncogenic mechanism, and the overexpression of miR-200b reduces its tumor suppressive activity by regulating ZEB1, which is relevant [46]. The level of editing of miR-200b has been described as inversely correlated with the expression of miR-200b, and it has also been associated with a worse prognosis than nonedited miR-200b [47]. In a related context, TTLL10-AS1, an autophagy-related long noncoding RNA, is part of a prognostic signature for ovarian cancer; its expression is associated with immune cell infiltration, PD-L1 levels, and chemotherapy sensitivity, ultimately influencing clinical outcomes [48]. Additionally, BEST1 promotes BC cell proliferation by facilitating calcium influx and activating the EGFR/AKT signaling pathway, highlighting its potential as a therapeutic target [49].

This study has several limitations related to the primary study. The identification of RNA edited sites within the cohort was performed via a previously reported methodology for detecting RNA editing sites [14, 50]. However, attempts to validate these findings in external cohorts were ineffective, likely because of differences in the preanalytic process and RNA sequencing methodologies used [11]. These discrepancies may have affected the detection of RNA-edited sites, limiting external validation in two trials of PARP inhibitors with rucaparib [51] and talozaparib [52]. Additionally, our models lack information on specific therapies received by each patient, complicating predictions for responses to individual drugs, as most cancer studies involve multiple drug combinations, even in clinical trials.

A probability risk score based on only 3 features could enable the development of a clinical tool to assess the risk of nonresponse to therapy. This tool could be implemented in daily clinical practice to guide clinical decisions when selecting personalized treatments and adjusting therapies according to each patient’s risk level. The identification of a few editing sites has the potential to be translated into clinical practice via RESqPCR, a modified PCR technique designed to detect specific edited sites [53]. This, in turn, could lead to significant improvements in outcomes for BC patients, such as higher response rates to selected treatments and a reduction in side effects associated with ineffective therapies. Furthermore, by identifying patients at high risk of nonresponse to certain therapies in a timely manner, it would be possible to explore alternative or more innovative treatment options, optimize resources and reduce the costs associated with prolonged or ineffective treatments. Similarly, this tool could help improve patients’ quality of life by reducing uncertainty about treatment effectiveness and avoiding unnecessary additional procedures.

## Conclusion

Our study emphasizes the potential of RNA editing as a valuable enhancement to predictive models for drug response in BC. By incorporating RNA editing alongside traditional omics data, including germline and tumoral DNA mutation data and RNA expression data, into machine learning models, we achieved high accuracy in predicting therapy response. The nonresponse risk score offers a practical tool for clinical application, providing a probability-based evaluation of therapy response. These findings indicate that integrating RNA editing into predictive models could advance personalized treatment approaches and support better decision-making in oncology.

## Supplementary Material

Supplement 1

## Figures and Tables

**Table 1:** Association of features with nonresponse to therapy

Chromosome: position	Gene name	Beta coefficient	p value	OR	95% IC OR	Point of index
Chr19:5111983	KDM4B	1.36	0.02	3.90	1.26–12.04	14
Chr1:1168162	MiRNA200/TTLL10-AS1	1.94	< 0.001	6.99	2.10–23.27	19
Chr11:61954112	BEST1	1.64	0.01	5.14	1.62–16.29	16

## Data Availability

The data used in this study were obtained from the publicly available dbGaP repository under the study accession number phs001050.v1.p1. The codes are deposited in https://github.com/ybernalg/RNA_editing_multiomic_machinelearning_models_breastcancer. Additional inquiries about the codes are available from the corresponding author upon reasonable request.
