# Supplementary material for: Integration of RNA Editing with Multiomics Data Improves Machine Learning Models for Predicting Drug Responses in Breast Cancer Patients
Source: Res Sq. 2024 Dec 17:rs.3.rs-5604105. Preprint. [Version 1] doi: 10.21203/rs.3.rs-5604105/v1 (PMC11702790; doi:10.21203/rs.3.rs-5604105/v1)
Supplement: Supplement 1 [file NIHPPRS5604105v1-supplement-1.pdf]

# Supplementary Files

This is a list of supplementary files associated with this preprint. Click to download.

- TablesSuppl.xlsx
- supppfigures.pdf
